# Supplementary material for: Risk factors for sacrococcygeal pilonidal sinus: a systematic review and meta-analysis supplemented by genetic causal assessment
Source: Front Surg. 2026 Jan 7;12:1718589. doi: 10.3389/fsurg.2025.1718589 (PMC12819706; doi:10.3389/fsurg.2025.1718589)
Supplement: Supplementary file 2 [file Datasheet2.zip › Supplementary Data 2/MR_pipeline_p5e-6/leaveone_plots/finn-b-E4_POCS.finngen_R12_L12_PILONIDALCYST.pdf]

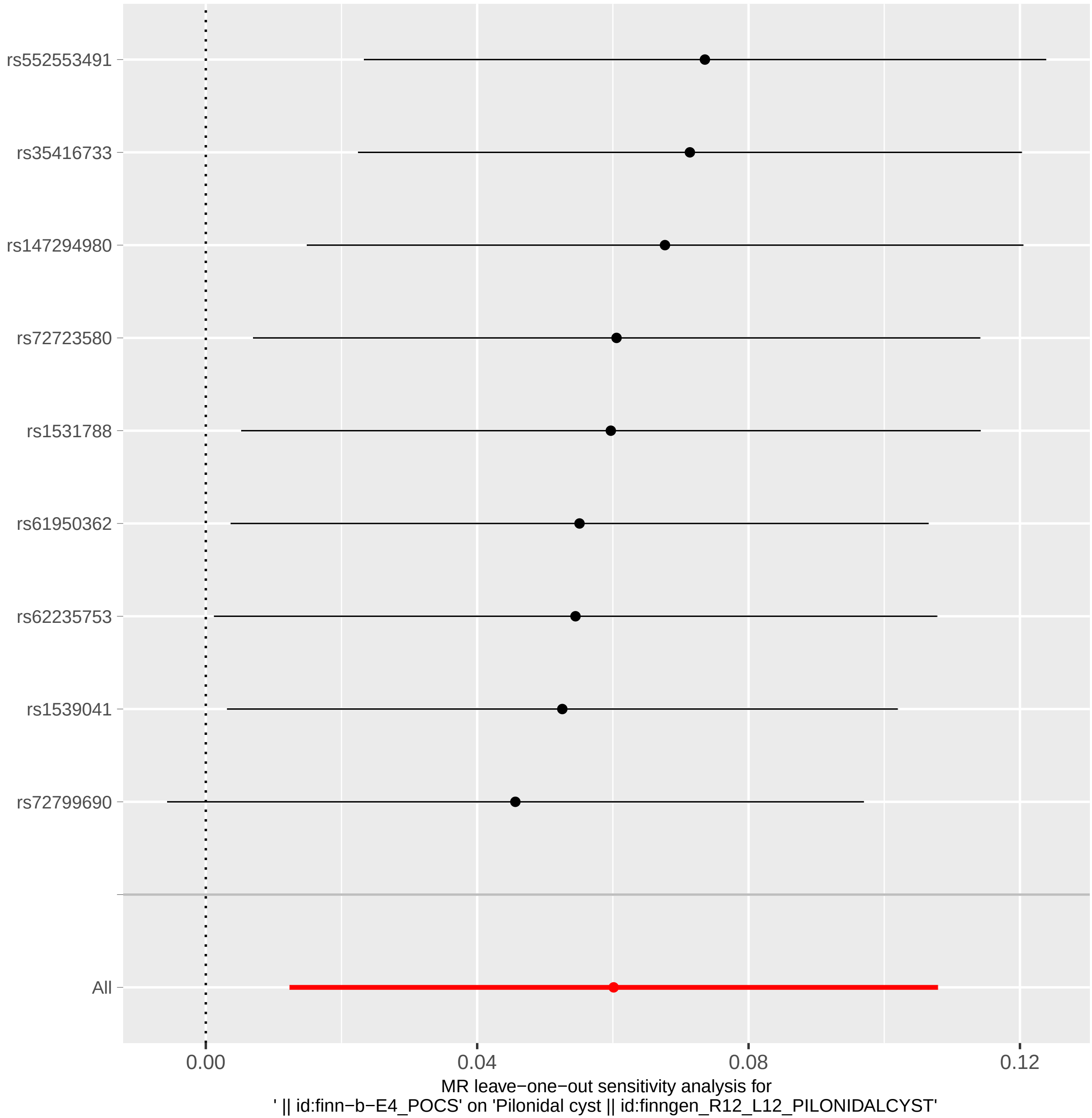
rs552553491
rs35416733
rs147294980
rs72723580
rs1531788
rs61950362
rs62235753
rs1539041
rs72799690
All
0.00
0.04
0.08
0.12
MR leave−one−out sensitivity analysis for
' || id:finn−b−E4_POCS' on 'Pilonidal cyst || id:finngen_R12_L12_PILONIDALCYST'
